# Identification of sibling species status of *Anopheles culicifacies* breeding in polluted water bodies in Trincomalee district of Sri Lanka

**DOI:** 10.1186/s12936-015-0726-z

**Published:** 2015-05-22

**Authors:** Nayana Gunathilaka, Prashath Karunaraj

**Affiliations:** Biotechnology Unit, Industrial Technology Institute, Colombo 7, Sri Lanka; Department of Chemistry, Molecular Biology and Biochemistry Degree Programme, University of Colombo, Colombo, Sri Lanka; Tropical Environmental Diseases and Health Associates, No 3 Elibank Rd, Colombo 5, Sri Lanka

**Keywords:** *Anopheles culicifacies*, Malaria, Vector

## Abstract

**Background:**

*Anopheles culicifacies s.l*., the major vector of malaria in Sri Lanka, is known to breed in clean and clear water. However, recent findings have confirmed breeding from waste water bodies in urban and semi-urban areas. No study has been conducted to identify whether it is vector or non-vector siblings. The objective of the study was to identify the sibling species status of *An. culicifacies s.l.*

**Methods:**

*Anopheles culicifacies s.l.* adult samples (reared from larvae) were obtained from the Padavisiripura Entomological team attached to Tropical and Environmental Diseases and Health Associates (TEDHA) Malaria Elimination Programme in Trincomalee District. The collected mosquito specimens were processed for the extraction of genomic DNA individually. The PCR amplifications were carried out using different primer combinations for differentiating species A from D, species B from C, species B from E, and species B, C, and E from each other. The results obtained from the gel electrophoresis were compared with the marker and band sizes of 359 bp, 248 bp, 95 + 248 bp, 166 + 359 bp and 178 + 248 bp were used to compare the sibling species A, B, C, D and E, respectively.

**Results:**

The molecular biological identification of the field caught *An. culicifacies s.l.* samples observed that only 13.34 % (4/30) was represented sibling species B. About 86.66 % (26/30) of the samples were *An. culicifacies* sibling species E. This study also provided evidence that *An. culicifacies* E was able to breed in a wide range of breeding habitats. This is the first time that *An. culicifacies* E breeding in waste water was confirmed by a molecular method. Malaria control programmes in most parts of the country focus on rural communities as a result of bio-ecology of mosquitoes. Therefore, unusual breeding habitats, such as waste water collections, may mislead the current vector controlling programmes.

**Conclusions:**

These results reconfirm that *An. culicifacies s.l.* has adapted to breed in a wide range of water bodies, including waste water collections. Since the majority of mosquitoes sampled belong to sibling species E, this may adversely affect the current malaria elimination programme and new strategies should be adopted to control malaria vectors breeding in these unusual breeding habitats in Sri Lanka.

## Background

*Anopheles culicifacies sensu lato (s.l.)* has a wide distribution in India, extending to Ethiopia, Yemen, Iran, Afghanistan and Pakistan in the west and Bangladesh, Myanmar, Thailand, Laos and Vietnam in the east. It is also found in Nepal and Southern China to the north and extends to Sri Lanka in the south [1, 2]. *Anopheles culicifacies s.l*. is the major vector of malaria in India and contributes for transmission of 60–70 % of the 2–3 million malaria cases reported every year [3]. It is also the best known and most important malaria vector in Sri Lanka [4]. It has thus far been recognized as a complex of five sibling species that are provisionally designated as species A, B, C, D, and E [5] and the distribution of these species has been mapped [6].

Out of all five, only sibling B and E has detected from Sri Lanka [4]. Sibling species complex B of *An. culicifacies* was considered responsible for malaria transmission in Sri Lanka [3]. However recently, the sibling species E has been recognized as the responsible vector for malaria transmission [7]. The sibling species exhibit distinct biological characters, host-feeding preference, biting activity, and susceptibility to commonly used insecticides in public health programmes [8].

Malaria control programmes in most parts of the country traditionally focus on rural communities, where the disease was prevalent. The bio-ecology of *Anopheles* breeding habitats in urban areas received very little attention [9]. Recent findings identified a conducive breeding of *An. culicifacies* in waste water containing drains in urban/semi-urban areas in Trincomalee District of Sri Lanka observing the highest larval density for *An. culicifacies* [9].

Since, current control activities target common types of breeding habitats having clear, sunlit, fresh waters such as sand pools, rock pools of drying rivers, margins of slow moving water and irrigation channels [10, 11], unusual breeding habitats are likely to be missed.

Therefore, identification of sibling species status is very important since the ability to transmit the malaria parasite is different from one sibling species to another [7]. Hence, accurate detection of sibling species status of malaria vectors may facilitate in developing cost effective vector controlling strategies under the current malaria elimination programme embarked in 2009 as a result of the significant reduction in malaria cases in the country.

Hence, the objective of the present study was to explore the sibling species status of *An. culicifacies* breeding in waste water habitats in order to adopt proper cost-effective vector control interventions through accurate vector identification.

## Methods

### Study areas

Malaria was considered as a major health problem for people living in rural agricultural communities in Sri Lanka and it was endemic in the Dry Zone of the country. The abundance of malaria vector, mosquitoes has not been studied in some parts of the Sri Lanka, especially in the Northern and Eastern Provinces over the past 30 years in view of the security situation. According to the recent publications, there are differences in the bio-ecology of anopheline mosquitoes [9]. Trincomalee district is an area where it showed some diversified breeding habitats for *An. culicifacies s.l.* The district of Trincomalee was selected for this study in order to determine the molecular identity of the main malaria vector, *An. culicifacies s.l.* breeding in unusual habitats.

### Selecting the sampling sites

**Fig. 1:**
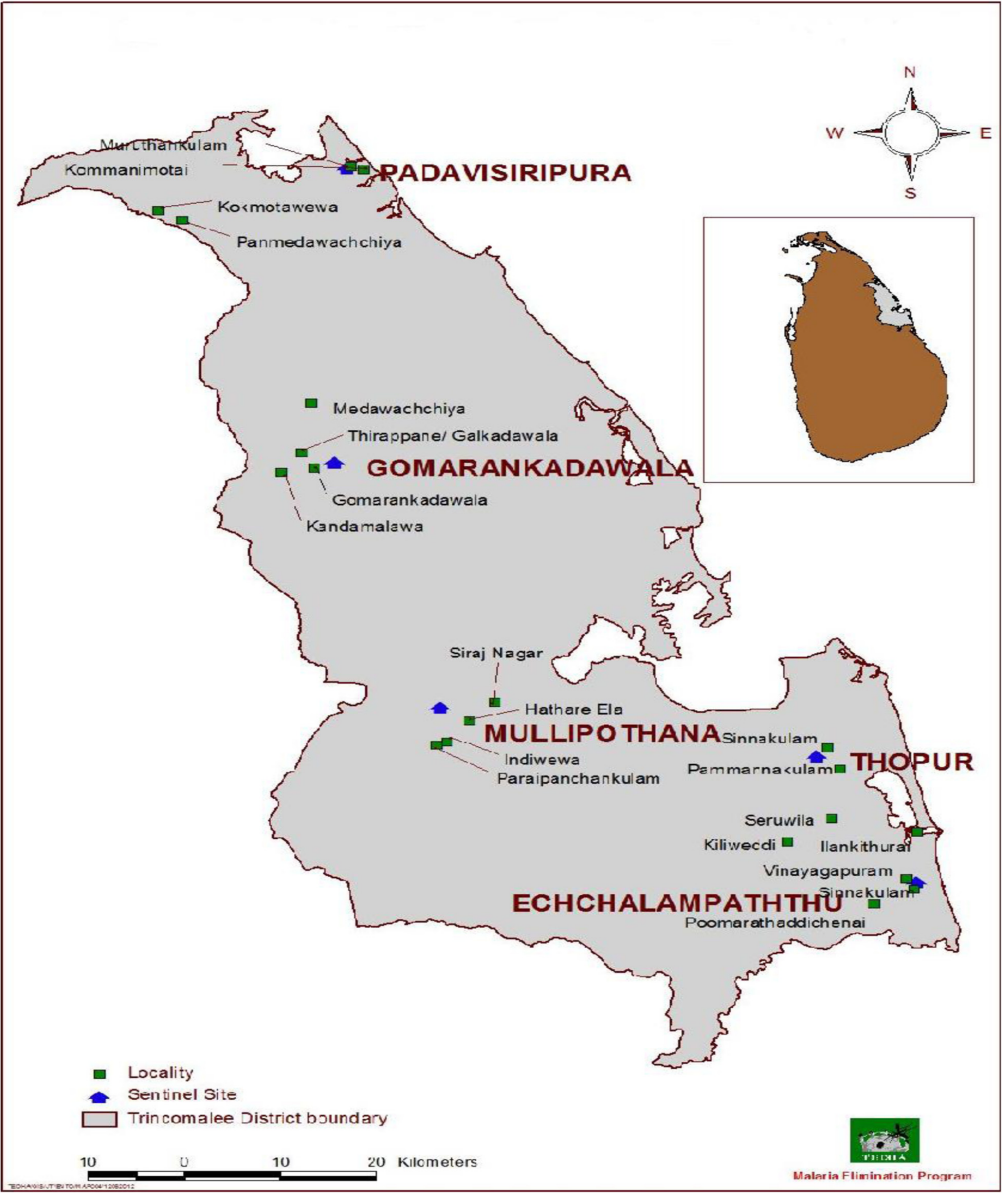
The map of Sri Lanka showing Padavisiripura and other sentinel sites in the District of Trincomalee

Out of five sentinel sites in the district of Trincomalee under the Malaria Elimination Programme of Tropical & Environmental Diseases & Health Associates (TEDHA) (Padavisiripura, Gomarankadawala, Mollipothana, Thoppur and Echchalampaththu), Padavisiripura site was identified as the area having more waste water habitats, which were more conducive for breeding of anophelines mosquitoes including *An. culicifacies s.l.* Therefore, the Padavisiripura sentinel site was selected for this case study (Fig. 1).

### Collection of *Anopheles* mosquito samples from the field

Anopheline mosquitoes from the identified waste water habitats were collected for a period of 3 months from March, 2014 to May, 2014 using standard dipping method as described by WHO [12].

### Larval survey

Mosquito larval specimens were collected using larval ladles [25 mm diameter, 250 ml capacity] with extensible handle. The number of dips was determined by considering the size of the breeding site. The number of dips and positive dips for *Anopheles* were recorded. Among the positive dips number of different larval stages were recorded (I-II and III-IV). The collected larvae were transferred to the field laboratory established at the Padavisiripura entomology office.

### Identification of field caught mosquitoes

Above collected larvae were reared in net covered plastic basins (30 cm diameter) filled with one litre of well water and feed with fish food dust. These larvae were reared until the adults emerged. Emerged adults were identified to the species level by using achromatic magnifying lens (× 10) and the standard morphological taxonomic keys prepared for Sri Lankan anophelines [13, 14].

### Processing mosquito samples

*Anopheles culicifacies s.l.* mosquitoes were separated from the adult mosquito collection obtained from larval rearing and individual specimens of *An. culicifacies s.l.* were separated into two parts, the head–thoracic region and the abdominal region. Both body parts were kept separately in two micro-centrifuge tubes. The processed samples were transferred to the Biotechnology Unit of Industrial Technology Institute, Colombo. The specimens were stored at −20 °C freezer until the DNA extraction.

### Extraction of genomic DNA

The genomic DNA was extracted from thirty specimens from both head-thoracic and abdominal regions of individual mosquito separately by phenol-chloroform method [15].

### Primer selection

The sequences of the mitochondrial *cytochrome oxidase-II* (*co-II*) gene for the five sibling species of the *An. culicifacies* complex (Table 1) were targeted as the PCR amplification region [16].

**Table 1:** Sequences of species specific primers used for multiplex PCR protocol

Name of the primer	Sequence (5’ to 3’)
ADF	CTAATCGATATTTATTACAC
ADR	TTACTCCTAAAGAAGGC
DF	TTAGAGTTTGATTCTTAC
BCEF	AAATTATTTGAACAGTATTG
BCR	TTATTTATTGGTAAAACAAC
CR	AGGAGTATTAATTTCGTCT
ER	GTAAGAATCAAATTCTAAG

### Optimization of the multiplex PCR

Trial PCR amplifications were performed using different primer combinations for differentiating species A from D, species B from C, species B from E, and species B, C, and E from each other. The PCR conditions were optimized with respect to parameters such as MgCl_2_ concentration, primer concentration, *Taq* DNA polymerase concentration, and annealing temperature. After achieving a set of reaction conditions from which products of the correct size were generated from the corresponding primer pair and DNA combinations, an effort was made to minimize the number of PCR assays needed to differentiate all five members of the complex.

### Agarose gel electrophoresis

Agarose powder (Promega) was used for the preparation of gels. The 1.5 % agarose gels which stained with ethidium bromide (0.5 μg/ml) were prepared. Amplified PCR products were loaded with Promega lambda marker and gel electrophoresis was carried out at 150 mV for 40 min to get a good separation in the amplified products. Following the gel electrophoresis, the migrated DNA was visualized and documented using gel documentation apparatus and Image Lab 3.0 software protocol (GelDoc ™XR+).

### Validation of results

The results obtained from the gel electrophoresis were compared with the marker and band sizes of 359 bp, 248 bp, 95 + 248 bp, 166 + 359 bp and 178 + 248 bp were used to compare the sibling species A, B, C, D and E respectively.

### DNA sequencing

In order to confirm the results in the experiment, about 50 μl of amplified PCR products using species-specific primers and universal primers were sent to Macrogen, Korea (10 F, 254 beotkott-ro, Guemcheon-qu, Seoul, Republic of Korea).

## Results

### Optimization of multiplex PCR assay for AD- PCR

The amplifications were conducted for species specific primers using 25.0 μl of solution containing 1.0 μl each of ADF (10 μM), ADR (10 μM) and DR (10 μM), 7.5 μl of Master mix [10× PCR buffer + MgCl_2_ (15 mM) + dNTPs- all four types (2 mM)], 1.0 μl of *Taq* polymerase (2 U/μl), 11.5 μl PCR water and 2.0 μl of 40× DNA template. The cycling conditions were hot start at 95 °C for 5 min followed by 40 cycles each of denaturation at 95 °C for 40 s, annealing for 48 °C for 1 min, and extension at 68 °C for 1 min, followed by the final extension at 68 °C for 10 min. The expected fragment sizes in the multiplex AD-PCR assay are follows: species A, 359 bp; species D, 166 + 359 bp.

### Optimization of multiplex PCR assay for BCE-PCR

Trial PCR amplifications were conducted using 25.0 μl of solution containing 1.0 μl each of BCEF (10 μM), BCR (10 μM), ER (10 μM) and CR (10 μM), 7.5 μl of Master mix [10× PCR buffer + MgCl_2_ (15 mM) + dNTPs- all four types (2 mM)], 1.0 μl of *Taq* polymerase (2 U/μl), 10.5 μl PCR water and 2.0 μl of 40× DNA template. The cycling conditions were hot start at 95 °C for 5 min followed by 40 cycles each of denaturation at 95 °C for 40 s, annealing for 48 °C for 1 min, and extension at 68 °C for 1 min, followed by the final extension at 68 °C for 10 min. The expected fragment sizes in the multiplex BCE-PCR assay are follows: species B, 248 bp; species C, 95 + 248 bp; species E, 178 + 248 bp.

### Molecular identification

**Fig. 2:**
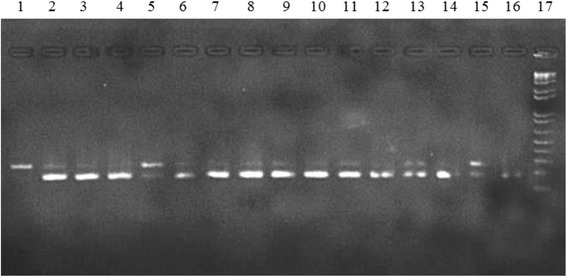
Multiplex BCE-PCR assay for the differentiation of sibling species status of the *An. culicifacies* complex. Lane 1: species B; lanes 2–16: species E; lane 17: 100-bp ladder (Promega)

The molecular biological identification of the field caught *An. culicifacies* samples observed that only 13.34 % (4/30) was represented sibling species B (248 bp). About 86.66 % (26/30) of the samples were *An. culicifacies* sibling species E (178 + 248 bp) which is considered as the vector for malaria (Fig. 2). None of the sample was positive for sibling species A, C or D.

### Validation of sibling species status of the field caught samples

**Fig. 3:**
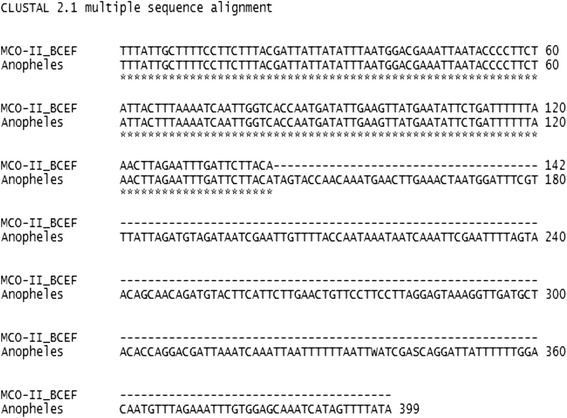
Clustal 2.1 multiple alignment of sequenced region of field caught sample with sequence of MCO-II region of *An. culicifacies* E available in NCBI

Sequenced region of PCR product (178 bp) of the field caught sample showed 100 % identical to the sequence of *mco-II* region of *An. culicifacies* E available in NCBI databank (Fig. 3). Since it has been proven that the samples collected from waste water breeding habitats showed 100 % identical to the malaria vector *An. culicifacies* E which is used to breed in clean water habitats, it is very important to develop a new vector controlling programme for malaria vector mosquitoes.

## Discussion

Accurate identification of anopheline mosquitoes is necessary for planning effective vector control strategies and for a better understanding of their potential role in malaria transmission [16]. Species identification based on morphological features is still practice by the field entomological teams. It must be emphasized still the use of morphological keys is an important and indispensable method for field identification of mosquitoes.

However, when comes to species complexes, they cannot be identified by morphological features, since they are morphologically similar. The importance of accurate identification of sibling species is, the ability to transmit the disease may vary from one sibling species to another. Therefore, it may adversely affect vector control efforts, if the vector is not correctly identified [17–20]. Hence, cytogenetical and molecular biological techniques are mandatory for a reliable identification of sibling species in a species complex.

A previous study conducted by Goswami *et al.* had used a multiplex BCE-PCR assay [16], while in the present study, the PCR amplifications were conducted for species-specific primers. Molecular assays targeting *internal transcribed spacer 2* (*its2*) and *co-II* genes are most commonly used in the identification of sibling species. However, the *its2* method has some limitations, since it can only differentiate sibling species into two sets as AD and BCE [21]. The *co-II* region does not have such drawbacks since this region is a conserved area. Therefore, this method can be easily used to differentiate the sibling species status undoubtedly.

Investigations on larval breeding sites suggests that species E is able to exploit a wide range of breeding habitats with different limnological characteristics [9]. There are some evidence in Sri Lanka to prove that the ability of anopheline mosquitoes breeding in waste water habitats with low dissolved oxygen level [14]. However, it was not characterized the sibling species status of the *An. culicifacies*. This is the first time the molecular status of *An. culicifacies s.l.* breeding in waste water habitats was characterized. According to the results, the majority of the species belongs to sibling species E, which is considered as the vector sibling species of *An. culicifacies*. This study also evidences that the ability of *An. culicifacies* E to breed in a wide range of breeding habitats. In addition, *Anopheles subpictus*, *Anopheles barbirostris*, *Anopheles peditaeniatus*, *Anopheles nigerrimus* and *Culex tritaeniorhynchus* species were also detected along with the *An. culicifacies s.l.* in these habitats.

There may be similar breeding habitats from other parts of the country, which allow breeding of anopheline mosquitoes. However, malaria control programmes in most part of the countries focus on rural communities, as a result of larval bio-ecology of anophelines. Therefore, unusual breeding habitats, such as waste water collections, may mislead the current vector control programmes. This investigation adds new information to the existing knowledge on malaria molecular entomology, as well as supporting more effective vector control programmes through accurate confirmation of vector species.

## Conclusions

The present study reveals, for the first time in Sri Lanka, the ability of *An. culicifacies* sibling species B and E breeding in drains containing waste water. It is suggested that health authorities should adopt new vector control interventions to eliminate these breeding habitats. This requires entomological surveillance in urban areas to detect potential vector breeding habitats.
